# The effects of genetic distance, nutrient conditions, and recognition ways on outcomes of kin recognition in *Glechoma longituba*

**DOI:** 10.3389/fpls.2022.950758

**Published:** 2022-08-17

**Authors:** Yilei Fan, Ruichang Zhang, Yuanlin Zhang, Ming Yue

**Affiliations:** Northwest University, Xi’an, China

**Keywords:** clonal plant, kin recognition, nutrient shortage, root exudates, genetic distance

## Abstract

Kin recognition might help plants decrease competitive cost and improve inclusive fitness with close genes; thus it might interact with environmental factors to affect communities. Whether and how various factors, such as the genetic distance of neighbors, environmental stressors, or the way a plant recognizes its neighbors, might modify plant growth strategies remains unclear. To answer these questions, we conducted experiments in which ramets of a clonal plant, *Glechoma longituba*, were grown adjacent to different genetically related neighbors (clone kin / close kin / distant kin) in different nutrient conditions (high / medium / low), or with only root exudates from pre-treatment in culture solution. By comparing competitive traits, we found that: (1) kin recognition in *G. longituba* was enhanced with closer genetic distance; (2) the outcomes of kin recognition were influenced by the extent of nutrient shortage; (3) kin recognition helped to alleviate the nutrient shortage effect; (4) kin recognition via root exudates affected only below-ground growth. Our results provide new insights on the potential for manipulating the outcome of kin recognition by altering neighbor genetic distance, nutrient conditions and recognition ways. Moreover, kin recognition can help plants mitigate the effects of nutrient shortage, with potential implications in agricultural research.

## Introduction

Kin selection benefits related genes and improves inclusive fitness; this altruism is favored in two ways: kin recognition and viscous populations (Hamilton, 1964). Most plants have limited dispersal, resulting in genetically structured populations within a small spatial scale (Karban et al., 2015; Ehlers et al., 2016; Anten and Chen, 2021), leading to a high likelihood of interactions with related neighbors, and making kin recognition important (Cheplick, 1992; Queller et al., 2015). Many previous studies provided evidence for kin recognition in plenty of plant species (Dudley and File, 2007; Murphy and Dudley, 2009; Biedrzycki et al., 2010; Masclaux et al., 2010; Bhatt et al., 2011; Biernaskie, 2011; Simonsen et al., 2015; Zhang et al., 2016; Xu et al., 2021), and that showed kin recognition can act as a driver not only always reducing some competitive traits (Dudley and File, 2007; Bhatt et al., 2011; Biernaskie, 2011; Crepy and Casal, 2015), but also sometimes increasing these competitive traits (Milla et al., 2009; Murphy and Dudley, 2009; Masclaux et al., 2010; Mercer and Eppley, 2014). Hence kinship might not be the only determinant in outcome of neighbor recognition, and other factors, e.g., nutrient conditions, water availability and/or other environmental stress, the way plants recognize kinship, or even the plant species, might affect the outcome of kin recognition. Thus more studies of how plants recognize relatedness are needed to understand this process more fully.

Previous studies have examined plant kin recognition among different related neighbors (Dudley and File, 2007; Murphy and Dudley, 2009; Biedrzycki et al., 2010; Bhatt et al., 2011; Mercer and Eppley, 2014; Semchenko et al., 2015; Abakumova et al., 2016), but there have been few studies to test how accurate the recognition might be and how different genetic distance affects kin recognition in specific species (Biedrzycki and Bias, 2010; Kiær et al., 2020). Depending on the dispersal mechanisms of a species, the nearest neighbors of clonal plants would be individuals of different genetic distances from that clone, including identical clones and kins with different degrees of relatedness (Ellstrand and Roose, 1987). Furthermore, crop species with artificially structured populations would have neighbors consisting of different related individuals of the same species (Murphy et al., 2017; Yang et al., 2018). Thus, plant of a given species might be favored by mechanisms that recognize and respond to different related neighbors more accurately (Dudley et al., 2013), so that they might avoid competition with the most closely related genes and so promote the survival of populations (Kiær et al., 2020). Overall, establishing whether plants are able to discriminate multi-level genetic distances and how they respond to different genetic neighbors from a same species might provide insights on how to modulate plant performance by adjusting the genetic structure in artificial plant populations, and suggest useful directions for further studies.

Since nutritional restriction is considered a dominant constraint on plant growth, density, and abundance (Tilman, 1984; Chapin et al., 1986; Ericsson, 1995; Bedford et al., 1999), some previous studies have focused on kin recognition under poor nutrient conditions, but the results have proved controversial. Recent research in *Sorghum vulgare* found that kin-benefit interactions in nutrient-poor soils were less pronounced than in nutrient-rich soil (Li et al., 2018). However, research in *Pisum sativum* showed plant kin selection was stronger in soil of lower fertility (Pezzola et al., 2020). A further study reported that the outcome of kin recognition changed several times when the growing distance from neighbors was altered from far to medium to close (Li et al., 2017). We can surmise that the two soil fertility studies found different consequences because they investigated kin recognition at two levels of fertility and their low fertility settings differed, and the response to kin varied under different degrees of nutrient shortage. Accordingly, we wished to investigate whether, under a range of nutrient decreasing conditions, the response to kin neighbors would remain constant or vary under different nutrient levels.

The capacity of plants to tolerate different constraining circumstances like nutrient shortage both above-ground (Smith, 1995; Anten, 2002; Falster and Westoby, 2003; Wang et al., 2014) and below-ground (Casper and Jackson, 1997; Maina et al., 2002; O’ Brien and Joels, 2008; McNickle et al., 2014) is primary for improving fitness, so it would be important if growing adjacent to genetically close neighbors might have benefits under unfavorable conditions. Some previous studies have tested whether plants can integrate both nutrient and neighbor stimuli and respond separately, yet the results were not clear (Gersani et al., 2001; Hess and Kroon, 2007; Cahill et al., 2010; De Kroon et al., 2012; Lamb et al., 2012; Padilla et al., 2013). For example, McNickle et al. (2016) found neighboring plants influenced root foraging performance more than nutrient conditions, which implied that the effect of plant–plant interactions on plant architecture was more important than the effect of nutrients. Because kin recognition always shows positive plant–plant interactions (Hamilton, 1964), we expected it might alleviate plant competition under low nutrient conditions, or mask the effects of small nutrient differences. In summary, the interactions between nutrient conditions and kin recognition remain unclear, and testing whether growing with nearby kins leads to benefits under unfavorable conditions might provide new insight into kin recognition.

Plant have evolved a variety of ways to acquire resources (light, nutrients, water, etc.) and to receive/emit signals from the environment, and root is the major organ of below-ground performance (Lal, 1979; Callaway and Mahall, 2007; Goebel et al., 2011; Depuydt, 2014). Root exudates are widely accepted as one of the most important mechanisms for below-ground interactions, and some previous research has focused on how root exudates mediate kin recognition (Biedrzycki et al., 2010; Mercer and Eppley, 2014; Semchenko et al., 2015; Wang et al., 2020). Previous studies showed that root exudates mediated kin recognition in *Arabidopsis thaliana* (Biedrzycki et al., 2010) and *Distichlis spicata* (Mercer and Eppley, 2014). But after recognition has occurred, the outcomes of kin recognition are different between these two studies. And it remains unclear whether it is root exudates affect the outcome of kin recognition both above and below ground. It has been reported that other factors, such as volatile chemical cues (Karban et al., 2015; Hussain et al., 2019) and photoreceptors (Crepy and Casal, 2015), were also able to mediate kin recognition. Thus, there might be various mechanisms by which plants can recognize neighbor identity, but there has been little research considering whether the specific outcomes of kin recognition might depend on the ways of recognition. Separating different potential ways of recognition, such as root exudates, would help us better understand how various mechanisms mediate kin recognition and influence its outcomes, and might provide new directions for future research on how responses to neighbors of different relatedness might be modified.

In the current study, we conducted a greenhouse experiment to explore kin recognition in a clonal plant, *Glechoma longituba*, and investigate how factors like neighbor genetic distance, nutrient stress, or ways of recognition affected plant growth. In our experiment, the plants had no initial below-ground parts, and the effects we detected as changes in plant architecture were most pronounced in the below-ground parts. Accordingly, we focused mainly on morphological traits below-ground and the relative growth rate (RGR) index of above-ground plant parts. Specifically, we tested four hypotheses: (1) kin recognition in *G. longituba* is stronger with closer genetic distance; (2) the outcome of kin recognition is influenced by nutrient shortage; (3) kin recognition helps to alleviate the effect of nutrient shortage; (4) kin recognition via root exudates can affect the growth of the whole plant.

## Materials and methods

### Experiments and plant materials

*G. longituba* is a normal stoloniferous clonal plant species in China. Each *G. longituba* ramet has two opposite leaves and produces roots when stolons touch the ground.

Genets of *G. longituba* used in our joint experiments were collected from Fenghuangzuigou (33.860 N, 108.825 E) and Hamagou (33.850 N, 108.818 E) in Qinling Mountains, which are 1.3 km apart with a valley between them. There were two different plots (1 × 1 m^2^) in Fenghuangzuigou which were more than 10 m apart and considered plot A_1_ and A_2_, while only one plot (1 × 1 m^2^) in Hamagou was considered plot B. We calculated the genetic distance of several genet samples collected from plot A_1_/A_2_ and plot B (Supplementary Figure 1). The analysis results showed genets from plot A_1_ has a closer genetic distance to genets collected from plot A_2_ than plot B, which demonstrates that genets from plot A_2_ can be considered as close kin to genets from plot A_1_ and genets from plot B can be considered as distant kin. Moreover, for clone species, fragment ramets from a same colon stolon would be same genetic identical but recognized as non-self to each other (Chen et al., 2015). So ramets from a same genet from plot A_1_ can be considered as clone kin to each other in our experiment. Then we have three clear genetic distance levels (clone, close, distant) of kin neighbor for genetic distance treatment. All genets were collected on 19th March 2016 and then planted in a greenhouse for 6 months before formal experiments. Ramets chosen in all experiments were seedlings that had not touched culture media so did not have below-ground growth at the beginning of our experiments.

**FIGURE 1:**
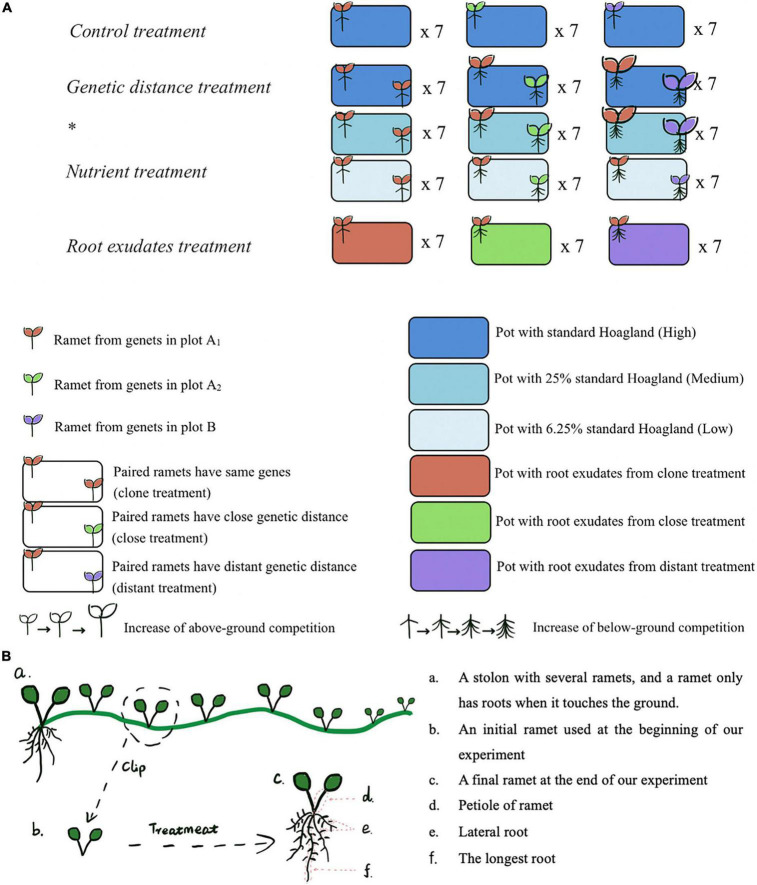
**(A)** Design of experimental units. After the Genetic distance treatment * Nutrient treatment, we kept the solution of the high nutrient level, which includes root exudates from ex-target and its neighbor, then put a ramet with the same gene of the ex-target in each pot as the *Root exudates treatment.* **(B)** A plant growth diagram showing the ramet material used in our experiment.

This joint experiment was designed to examine if *G. longituba* discriminates different related kins and how factors like genetic distance, nutrient stress, or the way plant recognizes its neighbor modified outcome of kin recognition (Figure 1A).

A control treatment was designed to make sure ramets used in our experiment from different plots (A_1_, A_2_, B) do not have significant difference in their growth performance (Figure 1A). And results showed the differences found in subsequent treatments were not caused simply by preexisting differences among ramets from the three plots (Supplementary Figure 2).

#### Genetic distance treatment * nutrient treatment

These two treatments were designed to verify if *G. longituba* can discriminate 3 levels of genetic distance and how genetic distance and nutrient stress influence appearance of kin recognition. To test this, we planted paired ramets in each pot and designed three different genetic-related levels: clone (the target plant and its neighbor sharing a pot were from a same genet from plot A_1_), close (target plant was from genets in plot A_1_ and its neighbor was from genets in plot A_2_), and distant (target plant was from genets in plot A_1_ and its neighbor was from genets in plot B) under a range of decreased nutrient levels (from high to medium to low). Each level had 7 replicates. Paired ramets were in similar size and the planting pots were full of high/medium/low nutrient solution (400 ml 100%/25%/6.25% Hoagland). The two ramets were placed at two diagonally corners of the pot to keep them separate during the experiment and avoid space competition. High/medium/low nutrient solution was added to each pot at the beginning and every 2 days during the experiment to maintain it at 400 ml (Figure 1A).

#### Root exudates treatment

This treatment was designed to examine how root exudates mediate kin recognition in *G. longituba.* The treatment was conducted in parallel with high nutrient condition group in Genetic distance treatment * nutrient treatment, kin recognition was supposed to be done after the previous treatment, thus when we removed the paired ramets, there would be liquid solutions left with 3 kinds of root exudates: exudates from target plant and its clone kin neighbor in clone pots, exudates from target plant and its close kin neighbor in close pots and exudates from target plant and its distant kin neighbor in distant pots. Each kind of pot had 7 replicates. Then after the Genetic distance treatment * nutrient treatment, we kept the old solution with root exudates in each pot and put one target plant in each pot. This target plant was from the same genet as the ex-target plant in this pot before. High nutrient solution was added to each pot at the beginning and every 2 days during the experiment to maintain it at 400 ml (Figure 1A).

### Growth conditions

The *G. longituba* ramets in our experiment were cultured in a greenhouse at 25°C during the daytime and 20°C during the night in summer 2018. In all experiments, ramets were fostered in pairs or individually in a pot (150 × 100 × 55 mm) with the corresponding culture solution (400 ml) for 10 days. During this time, the high nutrient solution (medium/low nutrient solution in the other two nutrient level treatments only) would be added in each pot every 2 days to constantly maintain the volume of growth medium at 400 ml.

### Measurement and statistical analysis

At the beginning and end of our experiment, fresh biomass, leaf area, and petiole length of each ramet were measured. And after experiments, length of the longest primary root, number of lateral roots were also measured. Then, specimens were separated into root, petiole, and leaf. These organics were dried at 60°C in an oven for 72 h and weighed separately.

Considering there was no initial below-ground of all ramets, we used one-way ANOVA to do multiple comparisons among groups and analyze the effects of neighbor relatedness on below-ground performance in all treatments, including root biomass, length of the longest root, and number of lateral roots (Figure 1B).

The ramets in our experiment had initial above-ground part (Figure 1B), so to decrease the effect of initial difference, we calculated RGR of fresh ramet biomass, leaf area, and petiole length by the following equation (Lugert et al., 2016).

RGR = (Wt-Wi)/Wi

where Wt is the final leaf area/petiole length, Wi is the initial/leaf area/petiole length. Then we used one-way ANOVA to do the *post hoc* test for these variables.

Then we used generalized linear mixed-effects modeling (GLMM) to test main effects of the two factors we focused on in our experiment (nutrient level and neighbor kinship), and their interactive effect on ramet growth performance we mentioned above.

All data were analyzed with SPSS 25.0 software. The data used in figures were all original data.

## Results

### Genetic distance and nutrient condition

Not only neighbor kinship has significant effects on ramet biomass traits and morphological traits (Table 1 and Supplementary Figure 3), but nutrient level also plays an important role here (Table 1 and Supplementary Figure 4). Moreover, there are significant interactive effects between these two factors on RGR of petiole length/leaf area, root biomass, and length of longest root. The two factors also have non-statistically significant interactive effect on ramet biomass and number of lateral root (Table 1).

**TABLE 1:** ANOVA results for effects on neighbor kinship (NK) and nutrient level (NL) on ramet biomass traits and morphological traits of *G. longituba.*

		Ramet biomass		RGR of petiole length		RGR of leaf area		Root biomass		Number of lateral root		Length of the longest root	
							
	df	*F*	*p*	*F*	*p*	*F*	*p*	*F*	*p*	*F*	*p*	*F*	*p*
NK	2	21.69	**0.000**	13.13	**0.000**	15.36	**0.000**	73.48	**0.000**	5.29	**0.007**	45.50	**0.000**
NL	2	5.46	**0.006**	6.58	**0.002**	7.27	**0.001**	20.40	**0.000**	11.63	**0.000**	7.85	**0.001**
NK*NL	4	2.15	0.084	3.91	**0.006**	5.89	**0.000**	8.87	**0.000**	2.04	0.098	7.26	**0.000**

Values for p < 0.005 are in bold.

**FIGURE 2:**
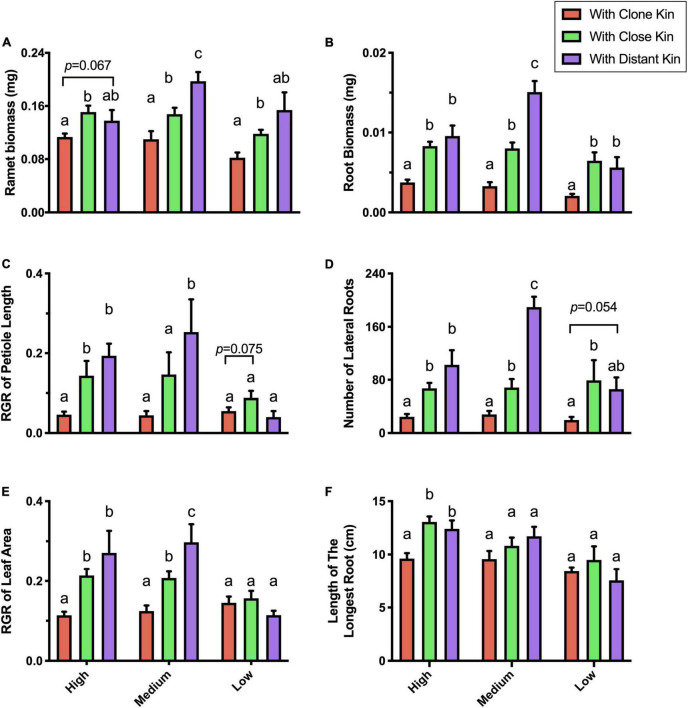
Competitive traits of *G. longituba* ramet response to different genetic related kins under different nutrient levels. **(A)** ramet biomass, **(B)** root biomass, **(C)** RGR of petiole length, **(D)** number of lateral roots, **(E)** RGR of leaf area, and **(F)** Length of the longest root. The different genetic related kins are either Clone Kin (target ramet and its neighbor have same genes), Close Kin (target ramet and its neighbor have close genetic distance) or Distant Kin (target ramet and its neighbor have the farthest genetic distance). The nutrient levels are high (100% Hoagland), medium (25% Hoagland), and low (6.25% Hoagland). Bars with different letters mean significant differences at *p* < 0.05.

Under the high nutrient treatment, *G. longituba* showed significant differences in most competitive traits between growing next to clone kin and growing with close or distant kin, while the difference between growing with close kin or distant kin was not statistically significant (Figure 2). Moreover, there was a general trend of increased investment in leaf, petiole, and root proliferation and bigger RII of roots when growing with more distant genetic kinship neighbors.

Under the medium nutrient treatment, *G. longituba* showed more obvious discrimination among kinship levels: there were significant differences among growing with clone kin vs. close kin vs. distant kin in all growth traits except root length, and the outcome of kin recognition was stronger than under the high nutrient treatment (Figure 2).

Under the low nutrient treatment, *G. longituba* showed little difference among the three types of related kins, and no clear kin discrimination was detected (Figure 2).

Nonetheless, *G. longituba* grown with clone kin showed the least change in most traits measured when the nutrient level was decreased, while ramets grown with close kin changed more and those grown with distant kin changed the most (Figure 2).

### Root exudates treatment

**FIGURE 3:**
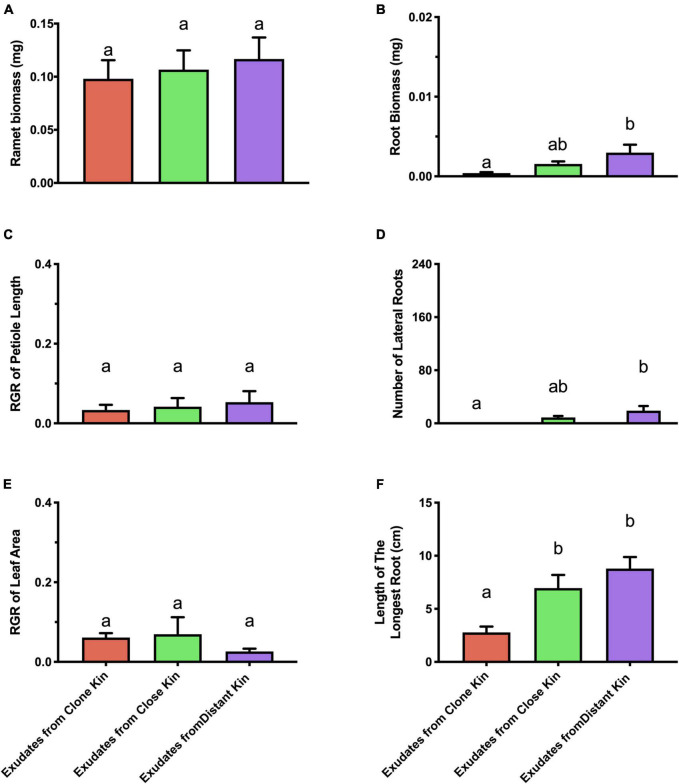
Competitive traits of solitary *G. longituba* ramets growing with root exudates from pre-treatment under high nutrient level. **(A)** Ramet biomass, **(B)** Root biomass, **(C)** RGR of petiole length, **(D)** number of lateral roots, **(E)** RGR of leaf area, and **(F)** Length of the longest root. Bars with different letters mean significant differences at *p* < 0.05.

Below-ground architectural traits of ramets in the root exudates treatment showed a similar trend to ramets grown under high/medium nutrient conditions in the genetic distance × nutrient level treatments (Figure 3). Both the RGR of petiole length, and leaf areas of target ramets in the root exudates treatment showed no significant difference among the three kinship levels (Figure 3), which suggested that *G. longituba* might have multiple ways to recognize neighbor identity, and the responses depended on the ways of recognition.

## Discussion

Our results confirmed the first three of our hypotheses, in that the experiments showed that the outcomes of kin recognition were modulated by the genetic distance of neighbors and the nutrient conditions. Specifically, although root exudates were able to mediate kin recognition, the responses depended on multiple factors, and root exudates mediated only below-ground outcomes of kin recognition.

Similar to most previous studies, *G. longituba* showed kin recognition with detectable outcomes both above and below ground under high nutrient conditions (Dudley and File, 2007; Murphy and Dudley, 2009; Biedrzycki et al., 2010; Masclaux et al., 2010; Bhatt et al., 2011; Biernaskie, 2011; Zhang et al., 2016). Moreover, the outcome of kin recognition appeared to be largely dependent on genetic distance. Decreased competitive abilities were correlated with increased relatedness of kin. We found *G. longituba* invested less in competitive morphological traits both above and below ground when the genetic distance between neighbors was closer. The lower RGR of leaf area and petiole length indicated less-successful competition for light (Smith, 1995; Gálvez and PeaCcy, 2003), while fewer root branches indicated reduced competitive ability in below-ground nutrient foraging (Biedrzycki et al., 2010; Semchenko et al., 2010; Marler, 2013). Overall, these results indicated reduced investment in resource acquisition and less competition between neighbors when they were closely related (Gersani et al., 2001). We also found that, although *G. longituba* competed less when the genetic distance of kin neighbors was closer, the discrimination between growing next to close kin and distant kin was not statistically significant. This might be because both the target plant and its neighbor were growing under favorable conditions, and the response to kin recognition might exert little effect on promoting population fitness; thus, while the plants might recognize different kins, this led to less-pronounced outcomes.

When the nutrient conditions were reduced from high to medium and even low levels, we found the outcome of kin recognition did not change in a pattern that correlated with nutrient levels. As mentioned above, there was no significant difference between *G. longituba* performance when growing with close kin or distant kin under high nutrient conditions; when the nutrient condition was decreased to medium, *G. longituba* statistically distinguished all clone/close/distant neighboring kins, revealing a stronger expression of kin recognition under the lower nutrient conditions. However, when nutrient levels were decreased to low, the outcome of kin recognition seemed to be masked by nutrient competition. This might explain why two recent studies on kin recognition and soil fertility found different results for plant performance with regard to kinship of neighbors under lower fertility (Li et al., 2018; Pezzola et al., 2020). The different results might be caused by different experimental designs for fertility, because our findings suggested the outcome of kin recognition was influenced by the extent of nutrient shortage. Another report showed similar results to ours, with similar outcomes for changes in kin recognition when the growing distance was altered (Li et al., 2017). Taking into consideration all these results, we speculate that the extent of environmental stress can affect kin recognition expression, and the expression changes from weak to strong to none when the stressor becomes more and more marked.

Because the response of *G. longituba* to kin recognition fluctuated under different nutrient conditions, we wondered whether growing next to closer kins might help plants improve their tolerance for low nutrient conditions. Many studies have shown that plants can synthesize information from their neighbors and nutrients and then respond to this information (Gersani et al., 2001; Novoplansky, 2009; McNickle and Brown, 2012; McNickle et al., 2016). A recent study in four grassland plant species found that information about neighbors exerted a stronger effect than nutrient levels in determining patterns of below-ground growth (McNickle et al., 2016), suggesting that kin recognition might play a dominant role in plant strategies when both neighbor identity and nutrient levels vary. In our research, both neighbor kinship and nutrient condition have significant effects on ramet biomass and morphology. Since these two factors also made significant interactive effects on most morphological traits, and growth of *G. longituba* was affected less by adjacent clone/close kins when grown under low nutrient conditions, indicating that growing with close genetic neighbors might buffer the effects of severe nutrient deficit. This result is similar to a previous study that examined kin selection in inter- and intra-specific competition, and suggested that kinship might influence plant growth (Mercer and Eppley, 2014). Generally, kin recognition is considered as a positive interaction, and kin neighbors are considered as a positive biotic factor by kin selection theory (Hamilton, 1964). Our study shows that kin recognition might play a dominant role in plant performance and help plants to tolerate poor conditions.

Previous studies have shown that root exudates can mediate identity recognition (Biedrzycki et al., 2010; Mercer and Eppley, 2014), and our research found *G. longituba* can recognize different genetic relatedness by ways of root exudates. But the effect was not exerted on the whole plant in our experiment, suggesting that kin recognition responses might be influenced by the method of plant recognition. In the genetic distance experiment, a target plant would not only have physical contact with its neighbor, but might also obtain information from other above-ground signals like volatile chemical cues (Karban et al., 2015; Hussain et al., 2019) and photoreceptors (Crepy and Casal, 2016). By contrast, in the root exudates experiment, there was no distant kin growing simultaneously in the growth chamber, and thus only one mechanism was available for kin recognition, namely the different root exudates from the pre-treatment. Our results showed that *G. longituba* was able to recognize different kins by root exudates alone, but if there were no other kinship signals, the response was exerted weakly only below-ground and not throughout the plant. The different response in above-ground growth between the genetic distance treatment and the root exudates treatment indicates that above-ground and below-ground signals modulated the kin recognition response together, and the overall outcome was the result of integrating multiple ways of recognition. In summary, *G. longituba* can recognize kinship by different mechanisms, and the responses are affected by those mechanisms; thus kin recognition is a complex and sensitive process (File et al., 2012; Crepy and Casal, 2016; Yang et al., 2021). The development of new technologies to monitor biotic/abiotic factors (Depuydt, 2014; Galieni et al., 2021) should enable further exploration of the effect of root exudates and other potential mechanisms of kin recognition.

In conclusion, our investigation of kin recognition in *G. longituba* revealed that individual plants can recognize intra-specific kinship levels more accurately than we thought before, and the outcome of that recognition is strongly influenced by nutrient conditions. Moreover, growing alongside close genetic neighbors might help plants mitigate the effects of nutrient shortages. There are diverse mechanisms by which plants recognize their kin; these mechanisms might also determine the response to kin recognition. Our findings suggest the outcomes of kin recognition could be manipulated in various ways, such as adjusting environmental conditions, selecting the kinship in populations, or controlling the ways plants recognize and respond to their neighbors. By studying kin recognition abilities in specific plant species in crops, ornamental plants, medicinal herbs, etc., we may make plants improve their investment in seeds, fruits, flowers, leaves, or other organs we need by taking control of different factors mentioned above. And benefits by growing with close kins could even improve plant tolerance under unfavorable conditions. Therefore, further studies on wider ranges of kinships, species, and other factors would help us better understand kin recognition in different plants, and new techniques would enable us to explore more details of kin recognition mechanisms, these relevant studies would provide new potential applications on agriculture, forestry, and environmental protection.

## Data availability statement

The raw data supporting the conclusions of this article will be made available by the authors, without undue reservation.

## Author contributions

YF performed the experiment and wrote the article. YZ analyzed the data. RZ revised the draft and finalized the manuscript for submission with YF and MY. All authors contributed to the article and approved the submitted version.
