# Supplementary figures and images for: The effects of genetic distance, nutrient conditions, and recognition ways on outcomes of kin recognition in Glechoma longituba
Source: Front Plant Sci. 2022 Aug 17;13:950758. doi: 10.3389/fpls.2022.950758 (PMC9428624; doi:10.3389/fpls.2022.950758)

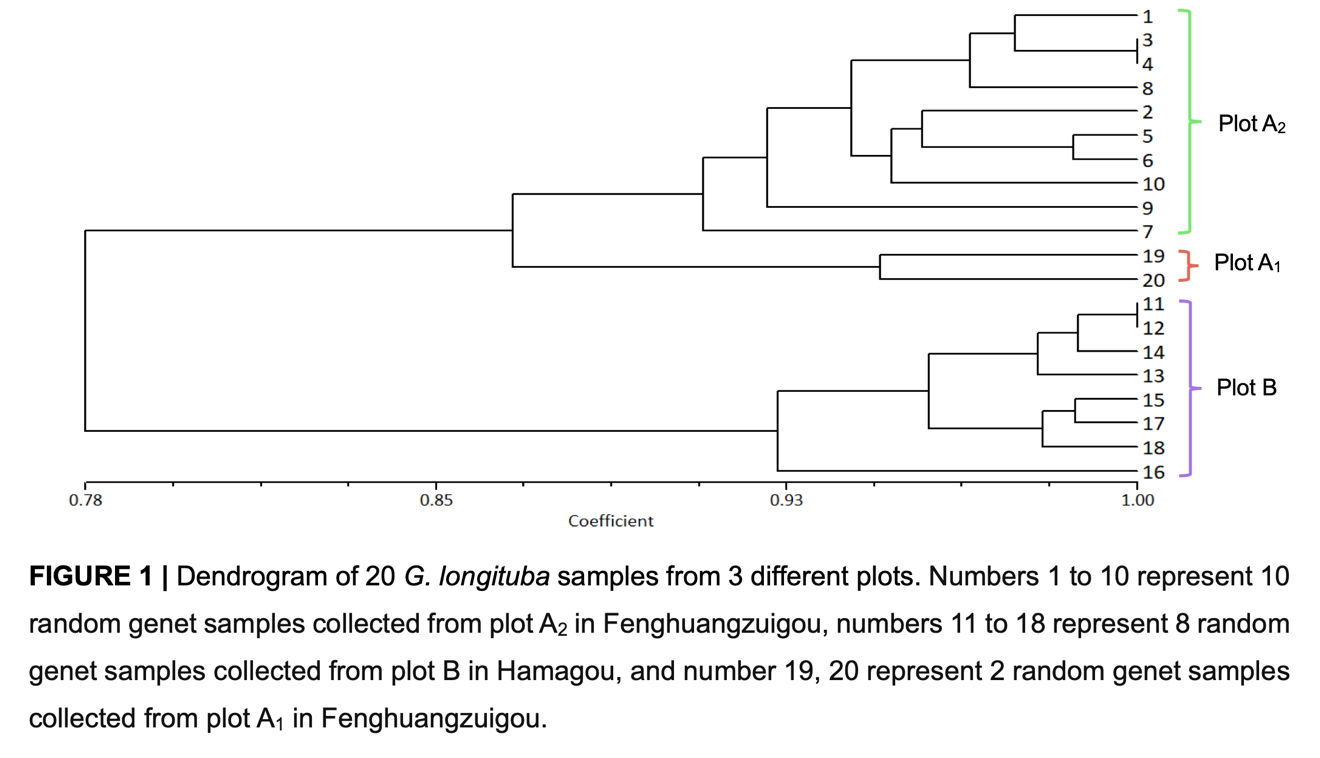

Supplement: Supplementary file 1 [file Image_1.png]

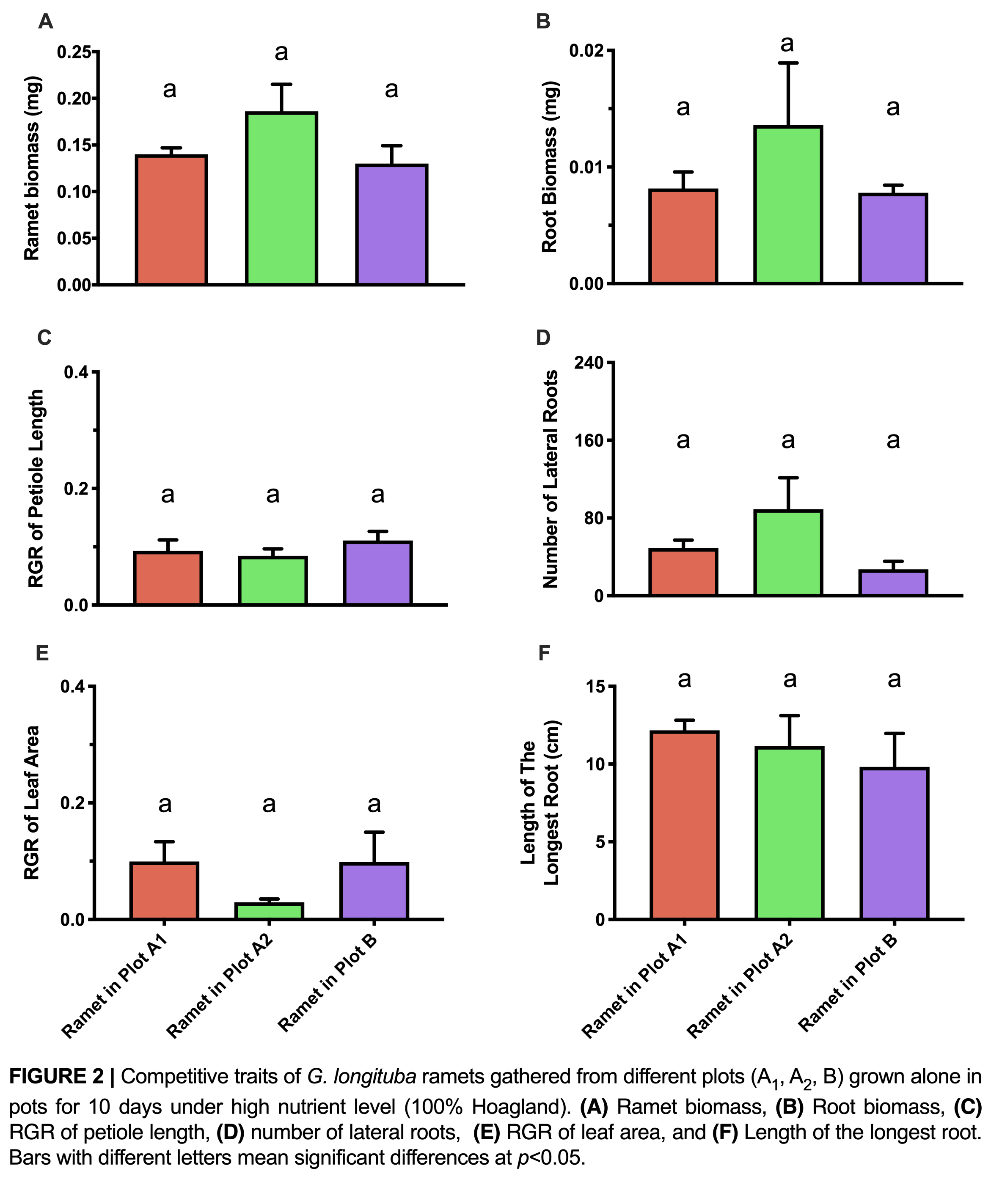

Supplement: Supplementary file 2 [file Image_2.tiff]

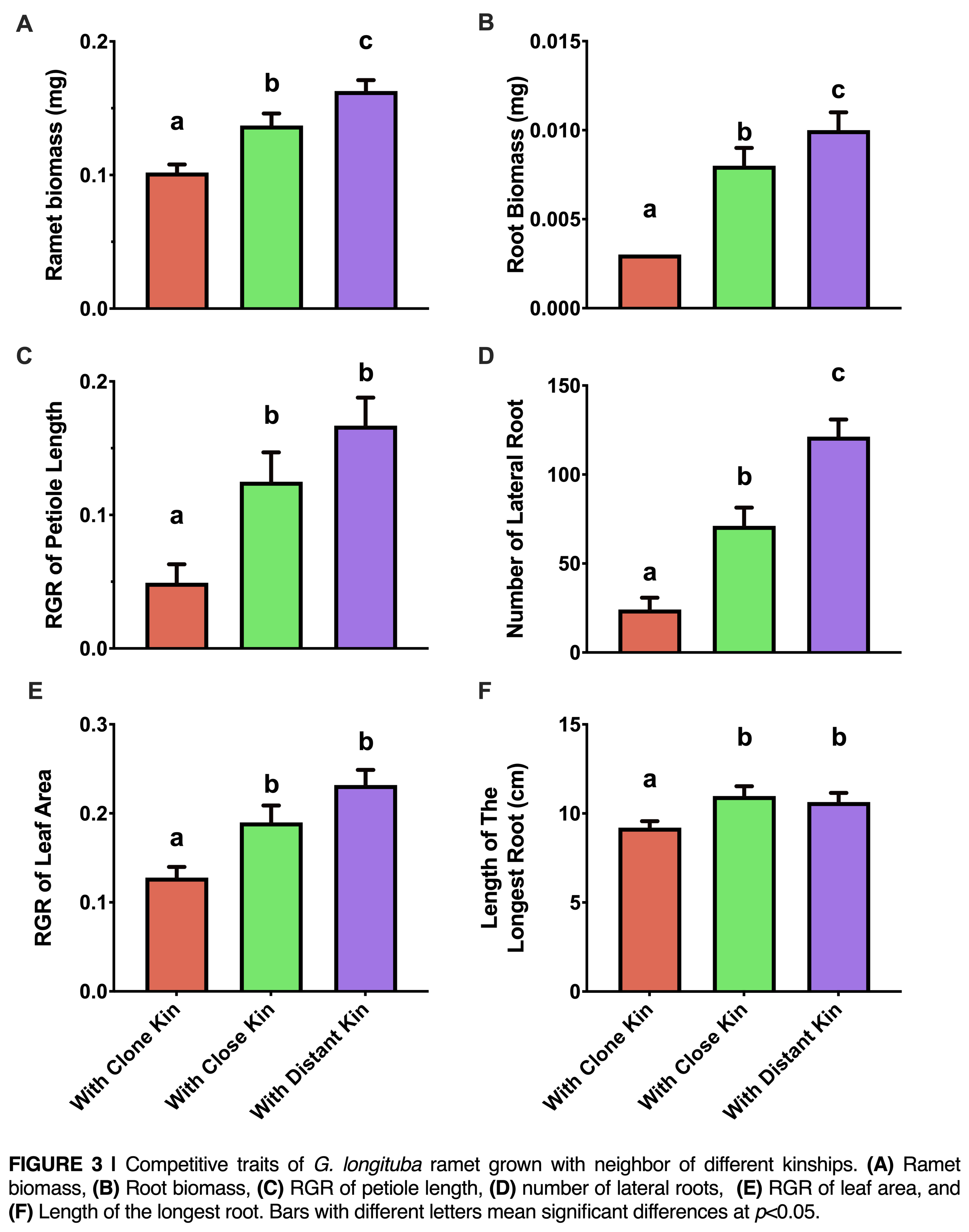

Supplement: Supplementary file 3 [file Image_3.tiff]

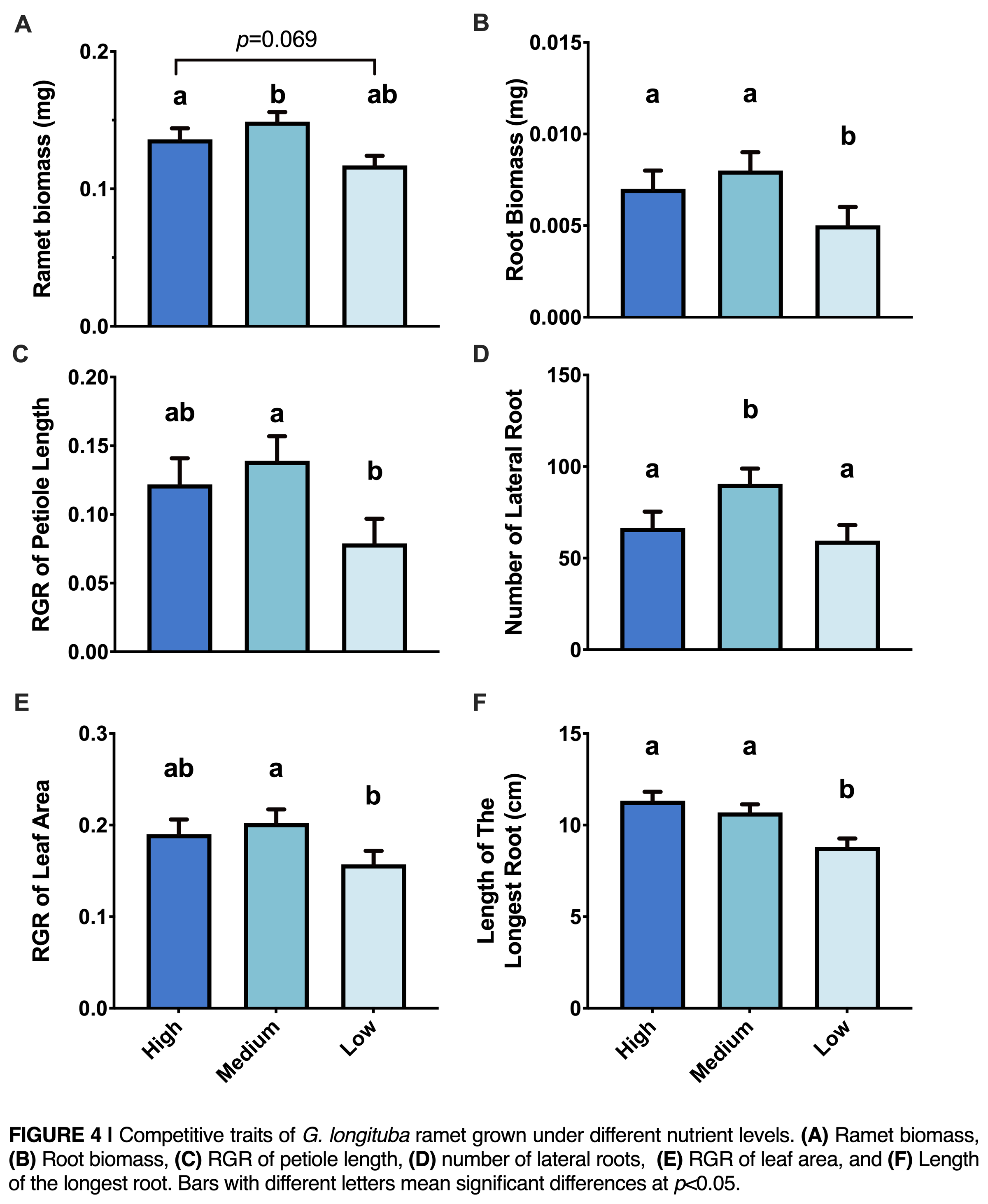

Supplement: Supplementary file 4 [file Image_4.tiff]
